# Non-Invasive Prenatal Screening from a Genetic Counseling Prospective: Pre and Post-Genetic Counseling Regarding Rare Chromosomal Abnormalities and Incidental Finding

**DOI:** 10.3390/genes15101349

**Published:** 2024-10-21

**Authors:** Della Monica Matteo, Cipriano Lorenzo, Piscopo Raffaele, Piscopo Carmelo

**Affiliations:** 1 Genetic Unit, A.O.R.N. “Antonio Cardarelli”, 80131 Naples, Italy; matteo191257@gmail.com; 2 Department of Molecular Medicine and Medical Biotechnology, University Federico II, 80131 Naples, Italy; lorenzo.cipriano@unina.it; 3 Eye Clinic, Department of Neuroscience, Reproductive and Odontostomatological Sciences, University Federico II, 80131 Naples, Italy; raffaele.piscopo@unina.it; 4 Medical and Laboratory Genetics Unit, A.O.R.N. “Antonio Cardarelli”, 80131 Naples, Italy

**Keywords:** prenatal screening, circulating cell-free DNA (cfDNA), non-invasive prenatal testing (NIPT), rare chromosomal abnormalities, incidental findings

## Abstract

Background: Arising in the late 1990s, when a promising role in prenatal diagnostics was first delineated for circulating fetal DNA, non-invasive prenatal tests (NIPTs) have been increasingly used with more frequency and popularity. These exams have been used as a prenatal screening tests for genetic diseases. Initially, they were developed for the investigation of the main fetal chromosomal aneuploidies, but lately they have also been used to rule out genomic microrearrangements and monogenic conditions. However, along with great opportunities and potential, the tests can show inconclusive or unexpected results. Several studies have shown that the current pre-test counseling is often insufficient, and more oriented at providing pieces of information about the identifiable diseases rather than providing extensive information on all possible scenarios which may affect both the fetus and the pregnant mother, especially in the case of an invasive test for the pregnant mother. Methods and Results: We have gathered from the literature on NIPT the main pitfalls, imperfections, and particular cases associated with this innovative diagnostic procedure. Conclusions: In view of further improvements in the methods that can limit the inconclusive or unexpected results, this paper aims to reinforce the importance of more accurate pre-test counseling with comprehensive information about the above-mentioned questions, as well as ultrasound use and also the creation of an international consensus statement concerning these topics.

## 1. Introduction

The presence of fetal cells in maternal blood has been an extraordinary discovery, and already reported way back in 1969 [1]. The chance of obtaining fetal cells during pregnancy generated great enthusiasm due to the possibility to identify, with a non-invasive approach, several fetal genetic anomalies. However, since then, many years have passed and considerable efforts had to be made, mainly in order to overcome the limited quantity of the methods available in recent years and also to address the limited quantity of the fetal material available for the diagnostics process. Eventually, in 1997, Lo et al. showed that cell-free DNA (cfDNA) could be reliably detected from maternal blood samples during pregnancy [2]. One year later, the same group demonstrated that a surprisingly high average concentration of fetal DNA (3.4–6.2%) could be found in total maternal plasma DNA [3]. The introduction of Next-Generation Sequencing (NGS) allowed great improvement in the study on DNA fragments of the entire genome or specific genomic areas. Since then, there have been numerous reports on the use of cfDNA for fetal chromosomal aneuploidies, mainly for trisomy 21 (Down syndrome), 18 (Edwards syndrome), and 13 (Patau syndrome), and sex chromosome anomalies. Shortly after, it was possible carry out further steps by using circulating cell-free DNA for the diagnosis of genomic microrearrangements [4], or even to identify monogenic hereditary conditions [5]. As usually happens with relevant scientific innovations, they carry great potential and perspectives but also limits and unexpected events. Indeed, the test’s reliability can be significantly affected by false positives (caused by placenta mosaicism, maternal mosaicism, statistical limit for positivity, maternal transfusions, or “resorption” of twins, also called vanishing twin syndrome, VTS), false negatives (due to placenta mosaicism, maternal transfusions, or insufficient quantity of total and/or fetal/placental DNA), unreproducible results (due to an insufficient quantity of total and/or fetal/placental DNA, autoimmune disease, heparin therapies, or “reabsorbed” twins), and incidental findings (maternal neoplasms or unexpected chromosomal anomalies) [6]. Therefore, placental, fetal, and maternal causes can affect the test. However, how can the results be interpreted, especially in the case of women testing positive for multiple chromosomal aneuploidy as a result of a suspected malignancy or if rare fetal autosomal trisomies are identified? How can a real and clinically reliable result be distinguished from an unreliable result due to placenta mosaicism? If it is simply an artifact or a placental mosaic, should the pregnant person just be reassured? If there is a well-founded suspect of a pregnant woman’s unexpected test result, what should be done in the presence of aneuploidies or copy number variations (CNVs) > 7 Mb, especially even in the absence of her explicit consent? The answer to these last questions should consider several technical, molecular, legal, and even ethical aspects, also depending upon different cultural, religious, and ethnic factors.

## 2. NIPT-Related Pitfalls and Particular Cases

### 2.1. Malignancy

Since around 90% of tumors gain or lose at least one chromosome arm, CNVs, including segmental/whole chromosome aneuploidies, are considered hallmarks of malignancies [7]. Both malignant and benign cancers can release cell-free neoplastic DNA into the maternal blood, confounding the NIPT findings. In general, a maternal cancer should be suspected when more than one aneuploidy, consisting of complex chromosome arrangements incompatible with a viable fetus, are detected. The detection of suspected, and unexpected, maternal malignancies raises complex medical, ethical, and psychological problems. Reporting the presence of a cancer during pregnancy needs to be carefully assessed, balancing the value of an early diagnosis of cancer and the risk of a false positive that can induce unnecessary stress about fetal and maternal outcomes. For this reason, it is necessary to establish a careful tradeoff between the best maternal diagnostic/therapeutic protocol and the fetal health safeguard, in particular providing an appropriate counseling pre-test. In this context, the possibility of detecting maternal cancer needs to be extensively explained as well as clarifying that, although some selected cancer therapies are currently considered safe during the second or third trimester of pregnancy, early chemotherapy treatment can affect the ability to carry the pregnancy to term [8]. Additionally, a post-test counseling provided by geneticists and oncologists could be a valid solution both to reduce the feeling of abandonment, commonly reported by mothers [8], and to perform a proper diagnostic investigation of the suspected tumor.

### 2.2. Placenta Mosaicism

As reported above, NIPT is still not considered a diagnostic test and invasive prenatal testing (e.g., amniocentesis and chorionic villus sampling) is always suggested for confirmation of chromosomal anomalies detected in pregnancy. This is mainly due to the high prevalence of false positives and false negatives [9]. The most common cause of false positives is confined placental mosaicism (CPM) [10,11], and cases of unaffected newborn with prenatal findings of aneuploidies have been previously described [12,13]. This is mainly related to the fact that the primary source of cffDNA in maternal circulation is the syncytiotrophoblast, and its aberrant products can falsify NIPT results. Chromosomal mosaicism is defined as the presence of two or more cell lines with different karyotypes in a single embryo and is a consequence of meiotic or mitotic nondisjunction errors. Abnormal cell lines can be found only in the fetus, only in the placenta, or in both of them. When the abnormal cell line involves both the placenta and the fetus or only the fetus, it is defined as true fetal mosaicism (TFM), whereas the CPM is a mosaicism isolated to the placenta. A CPM should be suspected in cases of low trisomic fraction relative to fetal fraction or when chromosomal aberrations incompatible with life are detected, especially without anomalies of the fetus at ultrasound exams. In cases of suspected CPM, amniocentesis represents the most reliable invasive prenatal testing, considering that it assesses DNA deriving from the amniocytes (i.e., of fetal origin), in opposition to the chorionic villus sample that evaluates placental cells. To prevent the overuse of prenatal invasive tests as a consequence of false NIPT results, positive testing should be integrated with prenatal biochemical screening tests and ultrasonographic markers, such as nuchal translucency, nasal bone presence, and several other useful structural anomalies, to improve the clinical interpretation of these findings. Suspecting CPM is also extremely relevant for the life of the fetus. In fact, the presence of CPM increases the risk of fetal growth restriction, small-for-gestational-age neonates, fetal loss, and preterm delivery, suggesting the need for specific intensified antenatal surveillance [14]. All this makes it necessary to provide to the parents detailed pre-test counseling on the risk of CPM and other false positives potentially (and wrongly) affecting the choice of termination of pregnancy. Potential needs to have to resort to invasive prenatal tests, with their related maternal–fetal complications, must be properly discussed and written informed consent must be obtained. In the case of a positive NIPT result, post-test counseling should be conducted by expert geneticists in the field of fetal medicine or by a multidisciplinary team composed of geneticists and gynecologists.

### 2.3. Copy Number Variations (CNVs) > 7 Mb

NIPT has been found able to detect copy number variants (CNVs) in several previous studies [15,16]. However, it suffers from a potentially much lower positive predictive value (PPV) for CNVs, microduplications, and microdeletions, resulting in a high incidence of false positives. Therefore, while this technique benefits from high performances in fetal common autosomal (trisomy 13, 18, or 21) and sex chromosomal aneuploidy detection, its utility in the field of CNVs is still a matter of debate. The relatively low PPV, high false-positive rate, and uncertain pathogenesis of CNVs raise problems in result interpretation, causing significant psychological stress on the mother, increasing unnecessary invasive diagnostic procedures (with the risk of severe adverse effects), and placing significant costs on the shoulders of the health system. For this reason, exhaustive pre-test counseling should be performed. In those cases, it should be properly explained that several CNVs remain of uncertain significance and that in most pathogenic/likely pathogenic CNVs it is impossible, in the prenatal period, to define the impact that a specific CNV will have on the clinical severity of the phenotype.

## 3. Discussion

Considering the extraordinary commercial success of the non-invasive prenatal test [17], its various opportunities, and the (relative) ease of performing it, as it involves a blood sample, it would seem that this test can avoid being under the traditional model of informed consent used for invasive tests. This test can be considered as not dangerous for pregnancy and it can easily provide various and considerable pieces of information. This concept entails the precise consequence that the amount of information provided to the woman and her husband can be considered little, so it can possibly be provided even by non-expert operators; moreover, it can be erroneously argued that the importance of the discussion is related to how extensive the investigation required may be, i.e., how many genetic diseases to test, providing information depending upon the reliability of the single test responses [18]. Indeed, the non-invasive prenatal test, as widely recognized by the international scientific community, is not a diagnostic test, but a genetic screening test [19], and it is therefore giving probabilistic results, carrying a margin of misleading information. Like any genetic test, it is strongly recommended by the guidelines and recommendations of several scientific societies that it should be offered through pre-test counseling [20]. The first essential point is therefore precisely this: the non-invasive prenatal test cannot lack an extremely accurate reconstruction of the family history of the attendant patients in order to avoid missing or underestimating risk conditions related to the patient’s personal details or clinical history [21]. The actual risk, in the absence of such an approach, is that, even if a broad test is chosen, this might not properly investigate a concrete potential reproductive risk or it may provide a result that is totally disconnected from the patient’s family history. Another important consideration is about the importance of an adequate attitude and professional preparation of the consultant, concerning which, once again as per the recommendations and guidelines, it is specified that “Prenatal genetic counseling associated with the test it must be provided by a specialist who has communication skills and competence and who uses a non-directive approach; so that the counselling represents a unique opportunity to clarify doubts and understand the implications, opportunities and limits of the analysis, so allowing the pregnant woman to be well informed about the choices” [20]. The information must be as exhaustive as possible and has to make the patient aware of possible discordant, insufficient, or unexpected results related to the woman’s health and the fetus’ well-being. It is therefore considered appropriate, after providing exhaustive information, to allow the woman to express her opinion about the possible need for further investigations [21], and to make the two attendants (the pregnant woman and her husband) aware about the possibility of discordant or rare results, such as those related to autosomal trisomies, possibly caused by placental mosaicism. In a 2023 statement [22] (Dungan et al. 2023), the American College of Medical Genetics and Genomics (ACMG) did not recommend using NIPT for autosomal aneuploidies, apart for those involving chromosomes 13, 18, and 21; the reason conveyed was that in some cases NIPT could be an unnecessary further economic burden, a factor increasing the anxiety of those being consulted, and a reason to resort to invasive prenatal fetal test procedures. Indeed, as per the literature, aneuploidies of many autosomal chromosomes are correlated with placental mosaicism which, when real, is associated very often with an increased risk, already in the first pregnancy trimester, of spontaneous abortion or intrauterine fetal death [23]. Studies in the literature [24] report that the overall probability of fetal DNA confirmation of rare trisomies is low or very low, reporting an overall positive predictive value for rare chromosomal abnormalities in a range from around 5 to 17% [25,26,27]. Therefore, the capacity of the non-invasive prenatal test to identify a true rare fetal trisomy is estimated to be approximately 1 in every 5000 pregnancy screening tests [20]. In these circumstances, a proposed flow chart [22] involves the execution of a careful evaluation of all ultrasound data, including the evaluation of the nuchal translucency measurement, an early careful examination of fetal morphology to detect any signs of fetal anomalies and malformations. The same study, however, specifies that it is not possible to exclude that in rare cases the fetus may have mosaicism, even in the absence of evident ultrasound anomalies. As a consequence, the indication for invasive prenatal diagnosis is justified in the case of ultrasound anomalies, but it cannot even be completely excluded without them. This viewpoint is also shared by the document of the Italian Society of Human Genetics/SIEOG/SIGO/AOGOI [28]. Among the most common rare aneuploidies is the chromosome 7 one, which is also very often due to a placental mosaic with a spectrum of fetal outcomes ranging from the absence of a clinical phenotype to reduced fetal growth [24]. The trisomy of chromosome 16 would seem to be more linked to an adverse outcome for the pregnancy, while in the case of aneuploidies of some chromosomes detected (6, 7, 11, 14, 15, or 20), the risk of uniparental disomies (UPDs) cannot be excluded. For the latter conditions, the indication for invasive prenatal diagnosis with the search for possible uniparental disomy would therefore be unavoidable [29]. In conclusion, for now, as advised from examining the data in the literature and the current indications of company documents, the clinical approach, although differentiated, must always be extremely prudent, by evaluating the accuracy of the process, the type of result, both the ultrasound and the pregnancy data, and also the attendants’ attitude towards the pregnancy. In Table 1, we summarize the most important NIPT-associated pitfalls and provide practical suggestions.

## 4. Conclusions

The genomic revolution has brought about extraordinary new developments and diagnostic possibilities. Among these, the study of cfDNA undoubtedly has represented an epochal turning point in prenatal diagnosis, offering an easy and very reliable pregnancy screening test, especially for the main fetal chromosomal aneuploidies. The test application has also led to several issues mainly related to the management of unexpected, discordant, or inconclusive results. The careful execution of pre- and post-test counseling, obtaining appropriate and exhaustive informed consent, and the concomitant use of ultrasound, which can be decisive in many cases, are of utmost importance. Additionally, in order to address doubts, provide reassurance, and save individuals from unnecessary anxiety or non-indicated recourse to invasive prenatal diagnosis, the further availability of increasingly greater and updated data on the outcomes of the NIPT results will ensure the right management of its use during the pregnancy. Further widely shared consensus statements and further validated data on the outcomes of pregnancies with NIPT anomalies would be highly beneficial to better guide the challenging decision-making process.

## Figures and Tables

**Table 1:** Summary of the NIPT-associated pitfalls and proposed solutions.

Special Circustances	Pitfall	Practical Suggestions
Maternal malignancies	Detection of unusual aneuploidies	Extensive pre- and post-test counseling
Rare autosomal trisomies	Low positive predictive (PPV) value due to confined placental mosaicism	Perform amniocentesis
Risk of uniparental disomy	Trisomy involving one of the autosomes that contain imprinted regions (6, 7, 11, 14, 15, and 20)	Perform amniocentesis
Copy number variations (CNVs)	NIPT’s reliability in this aspect depends on many factors such as the size of the CNV, the fetal fraction, and the sequencing depth resulting in low PPV	NIPT for CNV is not recommended for the routine care of unselected populations Expert post-test counseling
CNV < 7 Mb	Below the resolution of many current genome-wide NIPT platforms	Pre-test counseling declaring the impossibility of excluding CNVs below the resolution of the technique

## Data Availability

The original contributions presented in the study are included in the article, further inquiries can be directed to the corresponding author.
